# To Evaluate the Effect of Vitamin D on MicroRNAs in Polycystic Ovary Syndrome in Rat; An Animal Study: Effect of Vit-D on micro-RNAs in PCOS Rats

**DOI:** 10.31661/gmj.v14i.3759

**Published:** 2025-03-04

**Authors:** Fatemeh Attarian, Jina Khayatzadeh, Mohammad Mahdi Forghanifard, Saeedeh Zafar Balanezhad

**Affiliations:** ^1^ Department of Biology, Mashhad Branch, Islamic Azad University, Mashhad, Iran; ^2^ Department of Biology, Damghan Branch, Islamic Azad University, Damghan, Iran

**Keywords:** Polycystic Ovary Syndrome, Vitamin-D, Micro-RNA, Long Noncoding RNA, Testosterone

## Abstract

**Background:**

polycystic ovary syndrome (PCOS) is a metabolic disorder with menstrual and ovulatory irregularities and elevated risk factors for reproductive diseases that affects women in reproductive ages. A significant number of patients with PCOS have insufficient levels of vitamin- D (Vit-D). This study aimed to investigate the effect of Vit-D on the expression of micro-RNAs in PCOS in rats.

**Materials and Methods:**

24 female rats randomly were divided into four groups, including: 1) Control., receiving no drug (testosterone enanthate or Vit-D), 2) Pos., receiving the solvent of testosterone enanthate and Vit-D (ethanol), 3) induction of PCOS, receiving testosterone enanthate, and 4) Vit-D: treatment with Vit-D. The blood serum, used for the extraction of miR-186, mir-29, mir-21, Lnc ROR, Lnc MALA1, and H19 Lnc exosomes and the exosomes were separated from the serum. Data analysis was conducted using the ANOVA statistical test and SPSS software version 16 to examine significance at the level of P0.05.

**Results:**

The results showed that the expression of miR-186 was significantly increased in the Vit D group compared to the PCOS group (P0.01). Similarly, miR-21 expression was significantly higher in the Vit D group than in the PCOS group (P0.001). However, no significant difference was observed in miR-29 expression between the Vit D and PCOS groups (P0.05). Additionally, the expression of LncRNA H19 (P0.05), Lnc ROR (P0.001), and Lnc MALAT1 (P0.01) was significantly higher in the PCOS group compared to the Vit D group.

**Conclusion:**

These findings suggest that Vit-D plays a regulatory role in the expression of exosomal miRNAs and lncRNAs involved in PCOS pathogenesis. Given its potential to modulate genetic factors associated with PCOS, Vit-D supplementation could be considered as a supportive therapeutic strategy for managing PCOS. However, further studies are needed to explore its precise molecular mechanisms and clinical implications.

## Introduction

Polycystic Ovary Syndrome (PCOS) is a prevalent endocrine disorder affecting 8-11% of women of reproductive age, characterized by ovulatory dysfunction, hyperandrogenism, and polycystic ovarian morphology [1]. In Iran, its prevalence ranges from 7.1% to 14.6% [2]. PCOS is associated with metabolic complications such as insulin resistance, impaired glucose metabolism, dyslipidemia, hypertension, and cardiovascular disease [3]. It is diagnosed based on criteria from the National Institutes of Health (NIH), Rotterdam, or the Androgen Excess Society, incorporating hormonal imbalance and ovarian morphology [4].

Vitamin- D (Vit-D), a steroid hormone, plays a crucial role in reproductive function by influencing ovarian follicular growth, anti-Müllerian hormone signaling, and progesterone production [5]. Women with PCOS often exhibit Vit-D deficiency, which has been linked to increased androgen levels and metabolic disturbances [6]. Studies suggest that Vit-D may modulate molecular pathways involved in PCOS pathogenesis, including the regulation of microRNAs (miRNAs) and long non-coding RNAs (lncRNAs) [7].

Exosomes, small extracellular vesicles secreted by various cell types, play a key role in intercellular communication by transferring miRNAs and lncRNAs [8]. These molecules have been implicated in PCOS, with altered expression profiles observed in affected individuals [9]. miR-186, miR-21, and miR-29 regulate granulosa cell function, apoptosis, and steroidogenesis [10], while lncRNAs such as Lnc-ROR, Lnc-MALAT1, and Lnc-H19 influence ovarian function and metabolic pathways [11]. This study aimed to investigate the effect of Vit-D on the expression of micro-RNAs including miR-186, mir-21, mir-29 and Lnc ROR, Lnc MALA1 and Lnc H19 of serum exosomes in PCOS in rats.

## Method and Materials

**Figure-1:**
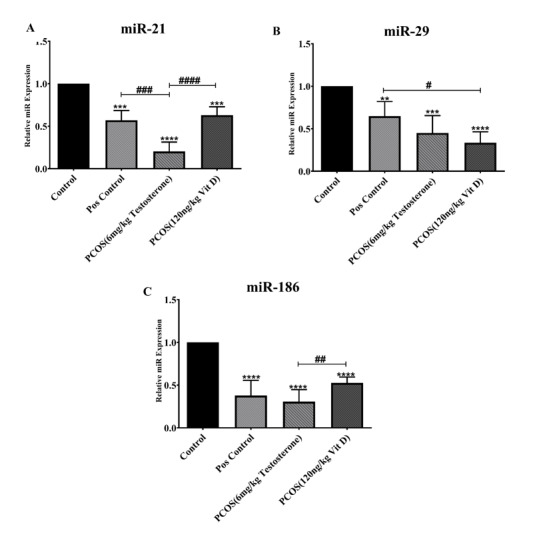
Figure 1. The relative expression of miR-21 (A), miR-29 (B) and miR-186 (C) in serum exosomes of PCOS compared to control and Vit-D groups. The graphs show measurement data, expressed as mean±standard deviation

**Figure-2:**
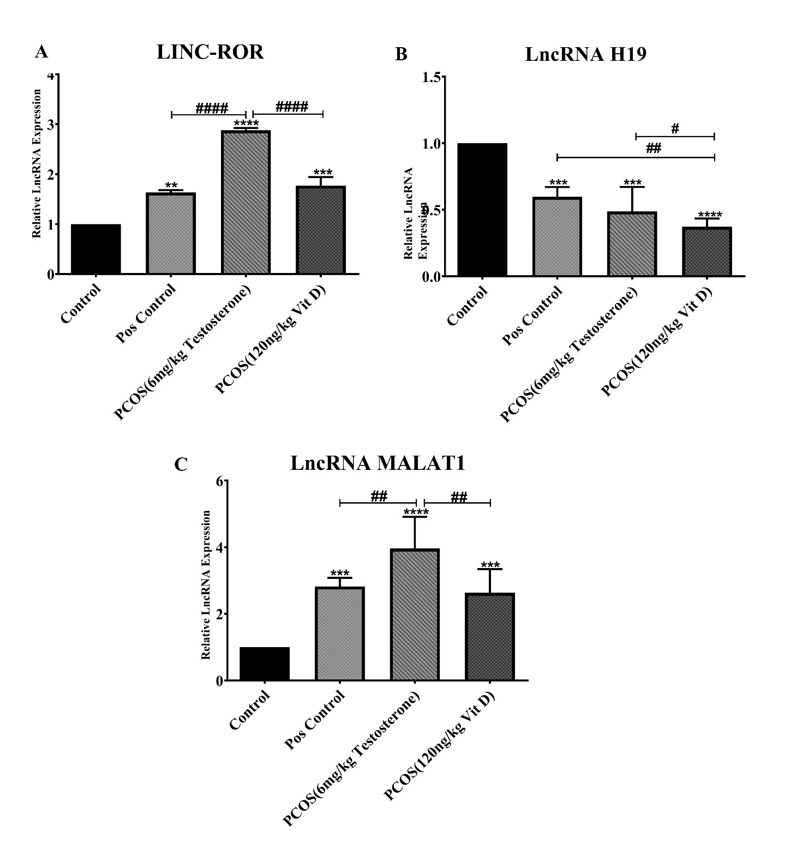
Figure 2. The relative expression of Lnc-ROR (A), Lnc-RNA H19 (B) and Lnc-RNA MALAT1 (C) in serum exosomes of PCOS compared to control and Vit-D groups. The graphs show measurement data, expressed as mean±standard deviation

**Figure-3:**
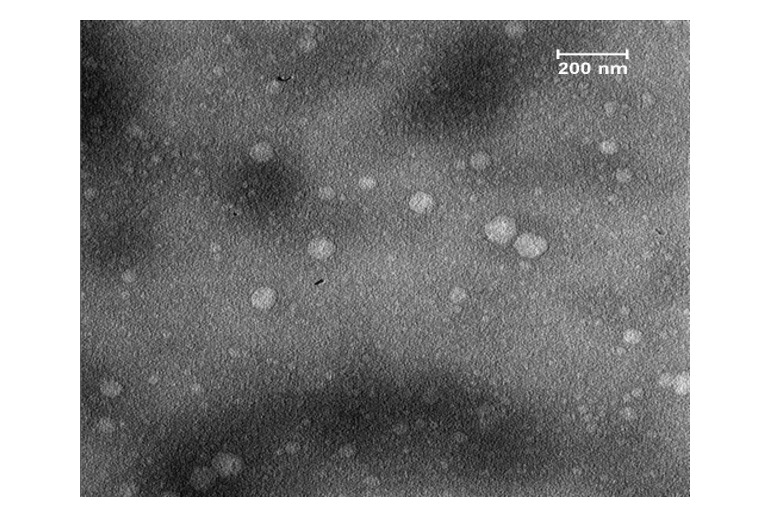
Figure 3. The TEM image of exosomes

24 prepubertal (21-day-old) female Wistar rats (250 ± 20 g) were randomly assigned to four groups (n=6) and housed under standard conditions (20-24°C, 12 h light/dark cycle, ad libitum food and water) at the School of Basic Sciences, Islamic Azad University, Mashhad. Rats with 2-3 regular estrous cycles over 12-14 days of vaginal smear observation were selected. The study was approved by the Institutional Animal Care and Use Committee (IACUC). Only the PCOS and PCOS + Vit-D groups developed polycystic ovary syndrome (PCOS), allowing comparisons between baseline (negative control), testosterone solvent effects (positive control), and PCOS with/without treatment [28][29][30].

### Vaginal Smear for Determining the Sexual Cycle

Vaginal smears were used to assess estrous cycle regularity. Rats with two to three consecutive regular cycles were selected. Cycle stages were determined by analyzing predominant cell types in daily vaginal smears from day 10 until the experiment's end [32].

### Induction of PCOS Phenotype

Several hormonal and non-hormonal methods, including testosterone, estradiol valerate (EV), dehydroepiandrosterone (DHEA), and adrenocorticotropic hormone (ACTH), can induce the PCOS phenotype. In this study, testosterone was used for hormonal induction in selected rats [30].

### Preparation of Testosterone Solution

PCOS was induced using testosterone enanthate (Iran Hormone Company), dissolved in sesame oil and ethanol, at a dose of 6 mg/100 g body weight (BW) for subcutaneous injection in the neck region over 35 days [30].

### Preparation of Vit-D Solution

Vit-D 3 (Iran Caspian Supply Company) was injected into rats at a specific dose (ng/g BW). To prepare the solution, 100 µl of Vit-D was mixed with 900 µl of sesame oil, followed by a second dilution with 100 µl of this mixture and 900 µl of sesame oil. The resulting solution was further diluted by combining 100 µl with 6.6 ml of sesame oil, reaching a final volume of 6.6 ml. Each rat received an injection of 20 units using an insulin syringe with the final solution.

### Animal Groups

Animals were randomly divided into 4 groups: (n=6)

1) Negative control group (entitled Control.): Receives no substance or drug (testosterone enanthate or Vit-D).

2) Positive control group (receiving testosterone solvent; ethanol) (entitled Pos.): Receives 0.2 ml sesame oil and 0.01 ml 95% ethanol subcutaneously in the back of the neck for 35 days.

3) PCOS group: PCOS induction by daily subcutaneous injections of 6 mg/kg testosterone for 35 days in the back of the neck [28].

4) PCOS group with Vit-D treatment (untitled Vit D): Injected intraperitoneally with 120 ng/g BW of Vit-D once a week for 5 weeks [30][33].

Instead of daily dosing, we used a weekly dose to reduce animal stress [29].

### Ovarian Tissue and Serum Sampling

Animals were euthanized under deep anesthesia using chloroform inhalation for ovarian extraction and histological studies. Blood samples were collected from the heart, and serum was obtained by centrifugation at 3000 rpm for 5 minutes. Serum, stored at -70°C, was used for miR-186, miR-29, miR-21, Lnc ROR, Lnc MALA1, and H19 Lnc exosome extraction. Ovaries were removed, fixed in 10% formalin, and tissue sections were stained with hematoxylin-eosin for microscopic examination [32].

### Exosome Extraction and Characterization from Serum

Exosomes were extracted from serum using the exoRibo™ Exosome Isolation Kit (Anasal) and separated via ultracentrifugation. Exosome characteristics, including morphology and diameter, were assessed using transmission electron microscopy (TEM) after fixation with 1% glutaraldehyde. The size and percentage of exosomes were determined by dynamic light scattering (DLS) using a Zetasizer instrument in a 100 μL phosphate buffer. This non-destructive, rapid, and cost-effective method quantified exosome diameter within the nanometer to micron range, confirming the isolation method [13].

### RNA Extraction and Real-time RT-PCR

mRNAs were extracted from ovarian tissues, and complementary DNAs (cDNA) were synthesized. Real-time PCR amplification was performed using a LightCycler and target gene primers (Macrogene, Seoul, Korea; Roche Diagnostics, Mannheim, Germany). Glyceraldehyde 3-phosphate dehydrogenase (GAPDH) served as the housekeeping gene [34]

### Data Analysis

Data were expressed as mean ± standard error. Statistical significance between groups was assessed using one-way ANOVA, with a significance level set at P<0.05.

### Ethics

IR.IAU.MSHD.REC.1401.042 (https://ethics.research.ac.ir/IR.IAU.MSHD.REC.1401.042)

## Results

**Figure-4:**
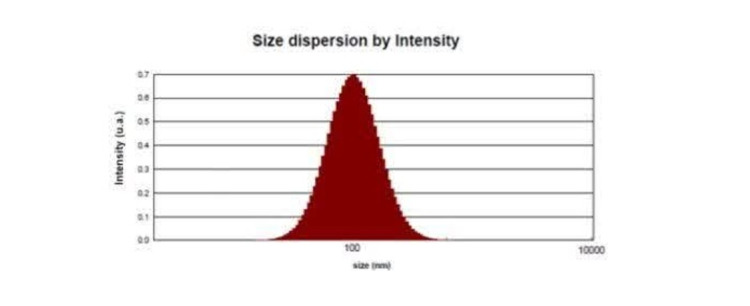
Figure 4. DLS analysis of exosomes

**Figure-5:**
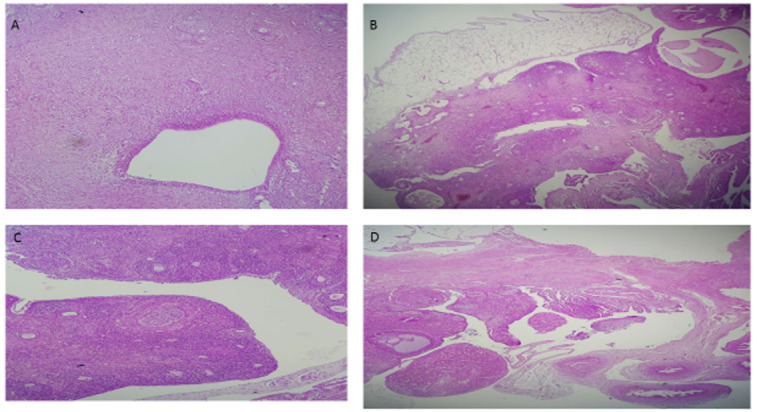
Figure 5. The histological image of ovarian sections in Neg. (part A), Pos. (part B), PCOS (part C), and Vit D (part D) groups.

### Effects of Vit-D on the Expression of miR-21, miR-29 and miR-186 in Serum Exosomes of PCOS

The relative expression of miR-21 (part A), miR-29 (part B) and miR-186 (part C) following the treatment with Vit-D are shown in the Figure-1. As it has been shown in part A, the expression of miR-21 significantly decreased in Pos., PCOS and Vit D groups compared to control (P<0.001). Treatment with Vit-D significantly increased the miR-21 expression in PCOS group (P<0.001). In addition, the miR-21 expression in Pos. groups significantly were higher than PCOS (P<0.001). The miR-29 expression (part B) was reduced in PCOS, Vit D (P<0.001) and Pos. (P<0.01) groups in comparison with Control. On the contrary, the miR-29 expression was elevated in Pos. compared to Vit D (P<0.05). Significant reduced miR-186 expression in Pos., PCOS and Vit D vs. control (P<0.001) is shown in Figure-1 part C. Treatment with Vit-D significantly increased miR-186 expression compared to PCOS (P<0.01).

### Effects of Vit-D on the Expression of Lnc-ROR, Lnc-H19 and Lnc-MALAT1 in Serum Exosomes of PCOS

To further explore the effect of Vit-D in PCOS, the relative exosomes expression was performed, which are shown in Figure-2. As it is depicted in Figure-2, part A, Lnc_ROR expression in PCOS (P<0.001), Vit D (P<0.001) and Pos. (P<0.01), was elevated vs. control group. PCOS induction significantly increased Lnc-ROR expression compared to Vit D and Pos. (P<0.001).

As it has been shown in Figure-2 part B, the expression of Lnc-RNA H19 significantly decreased in Pos., PCOS and Vit D groups compared to control (P<0.001). However, Lnc-RNA H19 expression in Pos. significantly was higher than Vit D groups (P<0.01). In addition, in PCOS Lnc-RNA H19 significantly was higher than Vit D (P<0.05).

Lnc-RNA MALAT1 expression is shown in Figure-2 part C. As it is shown Lnc-RNA MALAT1 expression was elevated in Pos., PCOS, Vit D groups (P<0.001) in comparison with Control. Induction of PCOS significantly increased Lnc-RNA MALAT1 expression in comparison with Pos. and Vit D groups (P<0.01).

### The Image of TEM

The exosomes that were obtained, as evidenced by the Transmission Electron Microscopy (TEM) image, were found to possess the typical characteristics associated with their kind (Figure-3). This includes a morphology that is considered standard for exosomes. Furthermore, their diameter was also within the expected range for such entities (30-150 nm) [36][37][38][39]. This information was derived from the TEM image, which provided a detailed visual representation of the exosomes. The image served as a reliable source for confirming the normalcy of the exosomes’ physical attributes.

### DLS Analysis Results

An analysis of exosome size was conducted using the Dynamic Light Scattering (DLS) method, and the results are depicted in the graph below (Figure-4). The findings revealed that the average size of the exosomes is approximately 139.56 nm, which was considered to be within the acceptable range for such vesicles. The graph also provides information on the hydrodynamic diameter, a measure that encompasses both the average particle diameter and the charge surrounding it. This diameter is reported to be 224.21 nm. Moreover, the Polydispersity Index (PDI), a metric used to gauge the distribution of particle sizes, is reported as 0.17 nm. This relatively low PDI value implies that the exosomes are dispersed uniformly and are monodisperse, suggesting a high level of consistency in their size distribution. Based on the data presented in the graph, it can be inferred that the exosomes display characteristics that align with their expected properties. These include a standard size range, a typical hydrodynamic diameter, and a uniform dispersion.

### Histology

The ovarian sections are shown in the Figure-5. The images demonstrate the histological data in Neg., Pos., PCOS, and Vit D groups in part A, B, C and D, respectively. In the Neg. and Pos. groups, the number of growing follicles is less than in the PCOS group, and the number of antral follicles and corpus luteum is greater than in PCOS. In the PCOS, the number of secondary growing follicles increased. Antral follicles and corpus luteum were fewer than in the Neg. and Pos. groups. In the Vit D, the number of secondary follicles is less than in the PCOS and close to the Neg. and Pos. groups. Additionally, the number of antral follicles is greater than in PCOS, and the number of secondary follicles is less than in the PCOS.

## Discussion

In the present study, the effects of Vit-D on the expression of exosomal miRNAs and lncRNAs were investigated in a rat model of PCOS. To investigate this, serum exosomes were extracted and the relative expression of miR-21, miR-29, miR-186 and Lnc-ROR, LncRNA H19 and LncMALAT1 in serum exosomes were measured. The results showed that the expression of miR-186 and miR-21 was increased in Vit-D -treated PCOS group compared to the PCOS. However, miR-29 expression were not notably reduced in PCOS. On the other hand, the expression of LncRNA H19, LncROR and LncMALAT1 was elevated in the PCOS compared to the Vit-D -treated PCOS group.

It is discovered that overexpression of serum miR-21 is found in PCOS and it is considered as a biomarker for PCOS diagnosis [21]. Other study showed that the serum levels of miR-21 circulation is higher in women with PCOS than healthy individuals [21]. In another study, the regulation of miR-21 and miR-186 and high DNA damage frequency is investigated in PCOS. According to this study increased DNA breakage frequency and miR-21 and miR186 expression levels in animals are indicative of chronic inflammation and oxidative stress, which are the primary causes of PCOS [40]. In the present study, induction of PCOS did not increase expression of miR-21 and miR-186 that is in contrary with these findings. But in another research, scientists investigated the dual role of miR-186 in cancer and found that the effects of miRNA dosage-dependent and frequency goals may help to understand the contradictory role of miR-186 in cancers [41].

So the different results in the present study can be attributed to the different doses. The role of miR-21 as a regulator of apoptosis and GCs proliferation in PCOS is investigated that showed the miR-21 is elevated in GCs of PCOSs [42], they also found a correlation between the expression levels of miR-186 in GCs and estradiol levels in women with PCOS, indicating that these two miRNAs could be used as new insights into the pathophysiology of PCOS [43].

The researchers evaluated the levels of miRNA in follicular fluid during IVF treatment in patients with PCOS (hyperandrogenic and normoandrogenic) and healthy individuals. The study showed that miR-29 decreased in follicular fluid of PCOS women since miR-29a-3p is associated with increased cellular growth by inhibiting homologous phosphatase and tenascin. Low levels of miR-29 potentially exacerbate the hormonal imbalance observed in PCOS by targeting two predicted target genes of miR-29 [44]. In another study, researchers induced PCOS in rats to confirm the molecular mechanisms underlying metformin in the intra-tissue environment. H19/miR-29b-3p/MMP-9 and H19/miR-29b-3p/MMP-2 signaling pathways were involved in PCOS, which was confirmed by metformin treatment of H19/miR-29b-3p and MMP-9/MMP-2/miR-29b-3p interactions [45]. These findings are in line with our results, as induction of PCOS following injection of Testosterone, decreased miRNA-29 expression in PCOS rats.

In the present study, the expression of LncRNA H19, LncROR and LncMALAT1 was elevated in the PCOS. These findings are in line with other studies that faced increased levels of Lnc in PCOS. For example, researchers assessed the plasma levels of MALAT1 as a biomarker for metastasis evaluation in epithelial ovarian cancer (EOC) in women with EOC by reverse transcription-quantitative polymerase chain reaction (RT-qPCR) to detect plasma MALAT1 levels. The comparison of results between this group and healthy women showed a significant increase [46]. In another study, scientists found that the expression of MALAT1 decreased in the ovarian tissue of PCOS rats. Overexpression of MALAT1 in laboratory conditions increases the proliferation and inhibits apoptosis of granulosa cells in the ovary. Overexpression of MALAT1 under in vivo conditions leads to a decrease in ovarian tissue damage and a decrease in FSH levels in PCOS mice. Overexpression of MALAT1 is associated with certain miRNAs [47].

Also,They found that metformin suppressed the expression of Lnc-H19 while increasing the expression of miR-29b-3p in a rat model of PCOS. Therefore, the role of metformin in PCOS was confirmed through the study of the activity of Lnc-H19 and AMPK signaling pathways in cell samples or serum collected from PCOS rats [45]. Other study showed that the expression of Lnc-H19 was significantly positively regulated in ovarian tissue of women with PCOS as well as GCs. Suppression of Lnc-H19 inhibited GC proliferation due to apoptosis stimulation [48]. In another study, researchers examined the expression levels of Lnc-ROR and miR-206 in the serum of individuals with PCOS to study the role and molecular mechanism of Lnc-ROR in PCOS. The results showed that the expression level of Lnc-ROR was increased in PCOS patients, while the expression level of miR-206 decreased compared to the control group [49]. In our study, miRs associated with PCOS prevalence decreased by treatment of Vit-D as it has been showed in some other studies [18][50][51][52][53]. Also, several studies have proved the effect of Vit-D on reduction of LncRNAs [54][55][56][57][58][59]. For instance Vit-D deficiency decreases Lnc- MALAT1 [58] and it is discovered that Vit-D regulates the expression of several lncRNAs [60] in consistent with our results which are showing the role of Vit-D in reducing LncRNAs . Generally, in PCOS, the interaction between Vit-D levels may be associated with the regulation of miRNA secretion by exosomes, but the exact underlying mechanism is not yet fully understood.

## Conclusion

These findings suggest that Vit-D may have a regulatory role in the expression of exosomal miRNAs and lncRNAs involved in the pathogenesis of PCOS. The downregulation of miR-186 and miR-21 may contribute to the development of PCOS, while the upregulation of miR-29, Lnc-ROR, LncRNA H19 and LncMALAT1 may be associated with the disease progression. Based on our findings, it can be concluded that Vit-D may be considered as an important factor in reducing the expression of genetic factors associated with PCOS. Moreover, it is possible that Vit-D can decrease the expression of genetic factors related to PCOS. However, further research and investigation are needed to elucidate the underlying mechanisms and potential therapeutic applications of Vit-D in PCOS.

## Conflict of Interest

There is no potential conflict of interest to declare.

## References

[R1] Huang X, et al (2020). Depletion of exosomal circLDLR in follicle fluid derepresses
miR-1294 function and inhibits estradiol production via CYP19A1 in
polycystic ovary syndrome. Aging (Albany NY).

[R2] Behboodi Moghadam, et al (2018). Polycystic ovary syndrome and its impact on Iranian women's
quality of life: a population-based study. BMC Womens Health.

[R3] Goodarzi MO, Carmina E, Azziz R, et al (2015). Dhea, dheas and pcos. The Journal of steroid biochemistry and molecular biology.

[R4] Huang P, et al (2021). Identification of three potential circRNA biomarkers of
polycystic ovary syndrome by bioinformatics analysis and validation. International Journal of General Medicine.

[R5] Matevossian K (2021). Polycystic ovary syndrome: menopause and malignancy. Clinical obstetrics and gynecology.

[R6] Walters KA, Allan CM, Handelsman DJ (2012). Rodent models for human polycystic ovary syndrome. Biology of reproduction.

[R7] TRIVAX B, AZZIZ R (2007). Diagnosis of Polycystic Ovary Syndrome. Clinical Obstetrics and Gynecology.

[R8] Mu Y, et al (2021). Vitamin D and polycystic ovary syndrome: a narrative review. Reproductive Sciences.

[R9] Hahn S, et al (2006). Low serum 25-hydroxyvitamin D concentrations are associated with
insulin resistance and obesity in women with polycystic ovary syndrome. Experimental and clinical endocrinology & diabetes.

[R10] Kuyucu Y, et al (2018). Investigation of the uterine structural changes in the
experimental model with polycystic ovary syndrome and effects of vitamin D
treatment: An ultrastructural and immunohistochemical study. Reproductive biology.

[R11] Aghadavod E, et al (2017). Evaluation of relationship between body mass index with vitamin D
receptor gene expression and vitamin D levels of follicular fluid in
overweight patients with polycystic ovary syndrome. International journal of fertility & sterility.

[R12] Chan BD, et al (2019). Exosomes in inflammation and inflammatory disease. Proteomics.

[R13] Ding SQ, et al (2020). Serum exosomal microRNA transcriptome profiling in subacute
spinal cord injured rats. Genomics.

[R14] Zhang X, et al (2015). Exosomes in cancer: small particle, big player. Journal of hematology & oncology.

[R15] Yu Y, et al (2021). MicroRNA-21 regulate the cell apoptosis and cell proliferation of
polycystic ovary syndrome (PCOS) granulosa cells through target toll like
receptor TLR8. Bioengineered.

[R16] Rusek AM, et al (2015). MicroRNA modulators of epigenetic regulation, the tumor
microenvironment and the immune system in lung cancer. Molecular cancer.

[R17] Balatti V, Pekarky Y, Croce CM (2015). Role of microRNA in chronic lymphocytic leukemia onset and
progression. Journal of hematology & oncology.

[R18] Li L, et al (2021). Exosomal miR-186 derived from BMSCs promote osteogenesis through
hippo signaling pathway in postmenopausal osteoporosis. J Orthop Surg Res.

[R19] Grive KJ (2020). Pathways coordinating oocyte attrition and abundance during
mammalian ovarian reserve establishment. Molecular Reproduction and Development.

[R20] Song Y, et al (2019). Altered miR-186 and miR-135a contribute to granulosa cell
dysfunction by targeting ESR2: A possible role in polycystic ovary syndrome. Molecular and cellular endocrinology.

[R21] Jiang L, et al (2015). Ciculating miRNA-21 as a biomarker predicts polycystic ovary
syndrome (PCOS) in patients. Clin Lab.

[R22] Naji M, et al (2017). Differential expression of miR-93 and miR-21 in granulosa cells
and follicular fluid of polycystic ovary syndrome associating with different
phenotypes. Scientific Reports.

[R23] Dalgaard LT, et al (2022). The microRNA-29 family: Role in metabolism and metabolic
disease. American Journal of Physiology-Cell Physiology.

[R24] Jiang H, et al (2014). Diverse roles of miR-29 in cancer. Oncology reports.

[R25] Sabol M, et al (2021). (In) distinctive role of long non-coding RNAs in common and rare
ovarian cancers. Cancers.

[R26] Qin L, et al (2019). Long non-coding RNA H19 is associated with polycystic ovary
syndrome in Chinese women: a preliminary study. Endocrine journal.

[R27] Zhang D, et al (2020). MALAT1 is involved in the pathophysiological process of PCOS by
modulating TGFβ signaling in granulosa cells. Molecular and cellular endocrinology.

[R28] Çelik LS, et al (2018). Effects of vitamin D on ovary in DHEA-treated PCOS rat model: A
light and electron microscopic study. Ultrastructural pathology.

[R29] Hadjadj L, et al (2019). Geometric, elastic and contractile-relaxation changes in coronary
arterioles induced by Vitamin D deficiency in normal and hyperandrogenic
female rats. Microvascular Research.

[R30] Noroozzadeh M, et al (2017). Hormone-induced rat model of polycystic ovary syndrome: A
systematic review. Life sciences.

[R31] Mohammad N, et al (2015). Effect of silymarin on estradiol valerate-induced polycystic
ovary syndrome. CABI Databases.

[R32] Sadoghi SD, Rahbariyan R (2017). Investigation the effect of glycyrrhizic acid on ovarian follicle
in polycystic ovarian syndrome mice model. Journal of Ilam University of Medical Sciences.

[R33] Przybylski R, et al (2010). Vitamin D deficiency in the spontaneously hypertensive heart
failure [SHHF] prone rat. Nutrition, Metabolism and Cardiovascular Diseases.

[R34] Asgharzadeh F, Attarian M, Khazaei M, Al-Asady AM, Mansoori S, Naimi H, Eskandari M, Khorrami A, Nazari SE, Aminian A, Farazastanian M (2025). Ziziphus jujube promotes fertility and pregnancy outcomes in Rat
model of uterine adhesions. Frontiers in Pharmacology.

[R35] Chen J, et al (2020). RNA profiling analysis of the serum exosomes derived from
patients with chronic hepatitis and acute-on-chronic liver failure caused by
HBV. Scientific Reports.

[R36] Li S, Chen L (2022). Exosomes in pathogenesis, diagnosis, and treatment of
hepatocellular carcinoma. Frontiers in Oncology.

[R37] Li MY, Liu LZ, Dong M (2021). Progress on pivotal role and application of exosome in lung
cancer carcinogenesis, diagnosis, therapy and prognosis. Molecular Cancer.

[R38] Zhu L, et al (2020). Isolation and characterization of exosomes for cancer research. Journal of Hematology & Oncology.

[R39] Mashouri L, et al (2019). Exosomes: composition, biogenesis, and mechanisms in cancer
metastasis and drug resistance. Molecular Cancer.

[R40] Salimi-Asl M, Mozdarani H, Kadivar M (2016). Up-regulation of miR-21 and 146a expression and increased DNA
damage frequency in a mouse model of polycystic ovary syndrome (PCOS). Bioimpacts.

[R41] Xiang Y, Tian Q, Guan L, Niu SS (2020). The dual role of miR-186 in cancers: Oncomir battling with tumor
suppressor miRNA. Frontiers in Oncology.

[R42] Yu L, et al (2021). Correlation between steroid levels in follicular fluid and
hormone synthesis related substances in its exosomes and embryo quality in
patients with polycystic ovary syndrome. Reprod Biol Endocrinol.

[R43] Song Y, et al (2019). Altered miR-186 and miR-135a contribute to granulosa cell
dysfunction by targeting ESR2: A possible role in polycystic ovary syndrome. Mol Cell Endocrinol.

[R44] Sørensen AE, et al (2016). MicroRNA Species in Follicular Fluid Associating With Polycystic
Ovary Syndrome and Related Intermediary Phenotypes. J Clin Endocrinol Metab.

[R45] Chen Z, et al (2019). Metformin treatment alleviates polycystic ovary syndrome by
decreasing the expression of MMP-2 and MMP-9 via H19/miR-29b-3p and
AKT/mTOR/autophagy signaling pathways. J Cell Physiol.

[R46] Chen Q, et al (2016). Plasma long non-coding RNA MALAT1 is associated with distant
metastasis in patients with epithelial ovarian cancer. Oncol Lett.

[R47] Chen Y, et al (2021). Down-regulation of MALAT1 aggravates polycystic ovary syndrome by
regulating MiR-302d-3p-mediated leukemia inhibitory factor activity. Life Sci.

[R48] Li L, et al (2021). Long non-coding RNA H19 regulates proliferation of ovarian
granulosa cells via STAT3 in polycystic ovarian syndrome. Arch Med Sci.

[R49] Zhang Z, et al (2021). Differential expression of long non-coding RNA Regulator of
reprogramming and its molecular mechanisms in polycystic ovary syndrome. J Ovarian Res.

[R50] Sheane B, et al (2015). An association between microRNA-21 expression and vitamin D
deficiency in coronary artery disease. Microrna.

[R51] Ross SA (2011). MicroRNA, Nutrition, and Cancer Prevention. Advances in Nutrition.

[R52] Liu PT, et al (2012). MicroRNA-21 targets the vitamin D–dependent antimicrobial pathway
in leprosy. Nature medicine.

[R53] Zhou Z, et al (2019). Vitamin D down-regulates microRNA-21 expression to promote human
placental trophoblast cell migration and invasion in vitro. Nan Fang yi ke da xue xue bao= Journal of Southern Medical University.

[R54] Chen S, et al (2017). H19 overexpression induces resistance to 1, 25 (OH) 2D3 by
targeting VDR through miR-675-5p in colon cancer cells. Neoplasia.

[R55] Shahrzad MK, et al (2021). Vitamin D and non-coding RNAs: new insights into the regulation
of breast cancer. Current Molecular Medicine.

[R56] Norouzi A, et al (2021). Exploring the expression profile of vitamin D receptor and its
related long non-coding RNAs in patients with acute lymphoblastic leukemia. Rev Assoc Med Bras (1992).

[R57] Kaur K, Allahbadia G, Singh M (2021). An Update on Long Non Coding RNAs as Prospective Targets for
Improving Prognosis of Colorectal Cancer by Acting as Biomarkers for Early
Detection of Metastasis, Getting Targeted for Inhibition of the MiaRNA they
Interact with that Promote Progession Along with Predicting Prognosis-A
Systemic Review. J Cell Mol Bio.

[R58] Nowrouzi-Sohrabi P, et al (2020). Vitamin D status influences cytokine production and MALAT1
expression from the PBMCs of patients with coronary artery disease and
healthy controls. Revista da Associação Médica Brasileira.

[R59] Gheliji T, et al (2020). Evaluation of expression of vitamin D receptor related lncRNAs in
lung cancer. Non-coding RNA Research.

[R60] Jiang YJ (2014). Lnc RNA: a new player in 1α, 25 (OH) 2 vitamin D3/VDR protection
against skin cancer formation. Experimental dermatology.

